# Differential Expression of CD163 on Monocyte Subsets in Healthy and HIV-1 Infected Individuals

**DOI:** 10.1371/journal.pone.0019968

**Published:** 2011-05-20

**Authors:** Emma Tippett, Wan-Jung Cheng, Clare Westhorpe, Paul U. Cameron, Bruce J. Brew, Sharon R. Lewin, Anthony Jaworowski, Suzanne M. Crowe

**Affiliations:** 1 Centre for Virology, The Macfarlane Burnet Institute for Medical Research and Public Health, Melbourne, Victoria, Australia; 2 Department of Medicine, Monash University, Melbourne, Victoria, Australia; 3 Infectious Disease Unit, The Alfred Hospital, Melbourne, Victoria, Australia; 4 Department of Immunology, Monash University, Melbourne, Victoria, Australia; 5 Department of Neurology, St Vincent's Hospital, Sydney, New South Wales, Australia; University of Rochester, United States of America

## Abstract

CD163, a haptoglobin-hemoglobin (Hp-Hb) scavenger receptor, expressed by monocytes and macrophages, is important in resolution of inflammation. Age-related non-AIDS co-morbidities in HIV-infected individuals, particularly dementia and cardiovascular disease, result in part from effects of HIV-1 infection on monocyte and macrophage biology. CD163 co-expression on CD14+CD16++ monocytes has been proposed as a useful biomarker for HIV-1 disease progression and the presence of HIV associated dementia. Here we investigated CD163 expression on monocyte subsets *ex vivo*, on cultured macrophages, and soluble in plasma, in the setting of HIV-1 infection. Whole blood immunophenotyping revealed CD163 expression on CD14++CD16- monocytes but not on CD14+CD16++ monocytes (*P* = 0.004), supported by CD163 mRNA levels. Incubation with M-CSF induced CD163 protein expression on CD14+CD16++ monocytes to the same extent as CD14++CD16− monocytes. CD163 expression on CD14++CD16+ monocytes from HIV-infected subjects was significantly higher than from uninfected individuals, with a trend towards increased expression on CD14++CD16− monocytes (*P* = 0.019 and 0.069 respectively), which is accounted for by HIV-1 therapy including protease inhibitors. Shedding of CD163 was shown to predominantly occur from the CD14++CD16− subset after Ficoll isolation and LPS stimulation. Soluble CD163 concentration in plasma from HIV-1 infected donors was similar to HIV-1 uninfected donors. Monocyte CD163 expression in HIV-1 infected patients showed a complicated relationship with classical measures of disease progression. Our findings clarify technical issues regarding CD163 expression on monocyte subsets and further elucidates its role in HIV-associated inflammation by demonstrating that CD163 is readily lost from CD14++CD16− monocytes and induced in pro-inflammatory CD14+CD16++ monocytes by M-CSF. Our data show that all monocyte subsets are potentially capable of differentiating into CD163-expressing anti-inflammatory macrophages given appropriate stimuli. Levels of CD163 expression on monocytes may be a potential biomarker reflecting efforts by the immune system to resolve immune activation and inflammation in HIV-infected individuals.

## Introduction

With the success of antiretroviral therapy in reducing the incidence of AIDS, attention is now turning to non-AIDS co-morbidities in chronically HIV-infected individuals. Two such important age-related co-morbidities that are accelerated in HIV-1 infection are cardiovascular disease and HIV-1 associated dementia, both of which involve activated macrophages and chronic inflammation [1], [2], [3], [4]. Biomarkers that detect persistent inflammation are needed to monitor immune activation and inflammation to enable better long term clinical outcomes for HIV-infected individuals. The scavenger receptor CD163 has been investigated as a potential marker of inflammation in infectious diseases such as meningitis [5], as well as in autoimmune diseases driven by activated macrophages [6], [7], [8], and so may have potential as a monitoring tool in management of HIV-1 disease.

CD163 is a member of the class B scavenger receptor cysteine-rich superfamily, primarily responsible for endocytosing hemoglobin-haptoglobin (Hb-Hp) complexes [9], [10] and, to a lesser degree, free hemoglobin (Hb) released from hemolysed erythrocytes [11]. Other more recently recognised functions of CD163 include anti-inflammatory scavenger receptor for the tumour necrosis factor-like weak inducer of apoptosis (TWEAK) [12], an erythroblast receptor, promoting their survival and differentiation [13], and a receptor functioning as an innate immune sensor for both Gram positive and negative bacteria [14]. CD163 expressing macrophages are also involved in resolution of inflammation by limiting free hemoglobin associated damage [11] and secreting anti-inflammatory cytokines in response to inflammation [15], [16]. However, the immune effects of CD163 are complicated and may not be limited to down-regulating inflammation as CD163 stimulation has also been reported to induce pro-inflammatory cytokine production from rat macrophages [17]. *In vitro*, soluble CD163, but not membrane bound CD163, inhibits phorbol ester-induced T cell proliferation [18], [19]. CD163 protein expression on monocytes and macrophages is altered in a number of diseases including asthma, cancer and following bypass graft surgery [20], [21], [22]. CD163 expression is increased by exposure of monocytes to glucocorticoids *in vivo*, and incubation with M-CSF, IL-6 and IL-10 *in vitro,* [23], [24], [25], [26], [27], [28] while it is decreased following incubation with pro-inflammatory cytokines GM-CSF, IL-4, IFNγ and TNFα [25], [27], [29].

CD163 is shed from the cell surface of monocytes following proteolytic cleavage after pro-inflammatory stimulation such as ligand binding (including lipopolysaccharide) to toll-like receptors [26], [30], [31], [32], [33], [34]. Soluble CD163 is thought to be involved in the resolution of inflammation by mechanisms that are not yet fully understood, but include inhibition of T cell activation [16], [18], and is increased in autoimmune disorders [32], [35], [36], hematological malignancies [37], malaria [38], and bacterial, but not viral meningitis [8]. Macrophage activation syndrome is driven by activated macrophages (and poorly functioning natural killer cells) and is associated with significantly increased plasma levels of soluble CD163 [6], [7]. Soluble CD163 levels in plasma correlate inversely with expression of CD163 on monocytes in blood obtained from randomly selected hospital patients [39] suggesting that monocytes are a major source of sCD163 under pathophysiological conditions.

In terms of HIV-1, productively infected macrophages and microglia in the brain of individuals with HIV-related encephalitis have been shown to upregulate CD163 [3], [40], [41]. In peripheral blood, the number of CD14+CD16++ monocytes expressing CD163 correlates with SIV RNA in plasma in SIV-infected rhesus macaques [40]. Levels of soluble CD163 in plasma of SIV-infected macaques correlate with monocyte activation and expansion [42]. In SIV-infected macaques with myocarditis, there was a correlation between CD163-expression on macrophages and the numbers of SIV-infected cells, but there were lower numbers of intra-cardiac CD163-expressing macrophages in SIV-infected macaques with myocarditis compared to controls, suggesting these cells are associated with decreased inflammation in the heart [43].

There have been contradictory reports on the expression of CD163 on different monocyte subsets with some studies reporting highest expression on the non-classical CD14+CD16++ monocytes [25], [44] while others report highest expression on CD14++CD16− monocytes [20]. CD163 is much more labile on monocytes than other surface molecules such as CD36 [45] and its expression on monocytes is altered both by the anticoagulant used in blood sampling and during purification of peripheral blood mononuclear cells [41], [45]. Recently it has been shown that inconsistencies in reported expression of CD163 on blood monocytes may also result from different sources of antibodies used for analysis, with epitope accessibility and CD163 detection being highest with antibodies directed against the N terminal of CD163 [46].

Given the proposed role of CD163-expressing monocytes in resolution of inflammation, and the potential for use of soluble CD163 as a marker for inflammation for HIV-infected individuals, we aimed to characterise CD163 expression on monocyte subsets and soluble levels in plasma in the setting of HIV-1 infection. We show that CD163 is more highly expressed on CD14++CD16- compared to CD14++CD16+ monocytes, and not expressed on CD14+CD16++ monocytes, at the level of both protein and gene expression, and that expression is increased in HIV-1 infection. Soluble CD163 in plasma was not significantly altered by HIV-1 infection. CD163 expression increased during maturation into macrophages and was inducible in all three monocyte subsets by culture in M-CSF.

## Materials and Methods

### Human research ethics

All human blood samples used in this study were collected with informed consent and approval from The Alfred Hospital Human Research Ethics Committee**,** project number 128/06 (Melbourne, Victoria, Australia).

### Sample donors

Peripheral blood was collected in K_3_EDTA tubes (BD Bioscience) from HIV-1 infected volunteers attending the Alfred Hospital Infectious Diseases Unit Outpatients (Melbourne, Victoria, Australia) or healthy HIV-1 uninfected laboratory volunteers. Controls were age matched to HIV-1 infected donors (HIV-1 negative median 40 yr vs. HIV-1 positive median age 42 yr). Table S1 describes the viral load, CD4 T cell count and antiretroviral therapy regimen of HIV-1 positive donors at time of recruitment. To harvest plasma, K_3_EDTA tubes containing blood samples were centrifuged at 620×*g* for 10 min with no brake. The uppermost 3 ml was removed and stored at −80°C until analysed.

### Whole blood flow cytometry phenotyping

**Figure 1:**
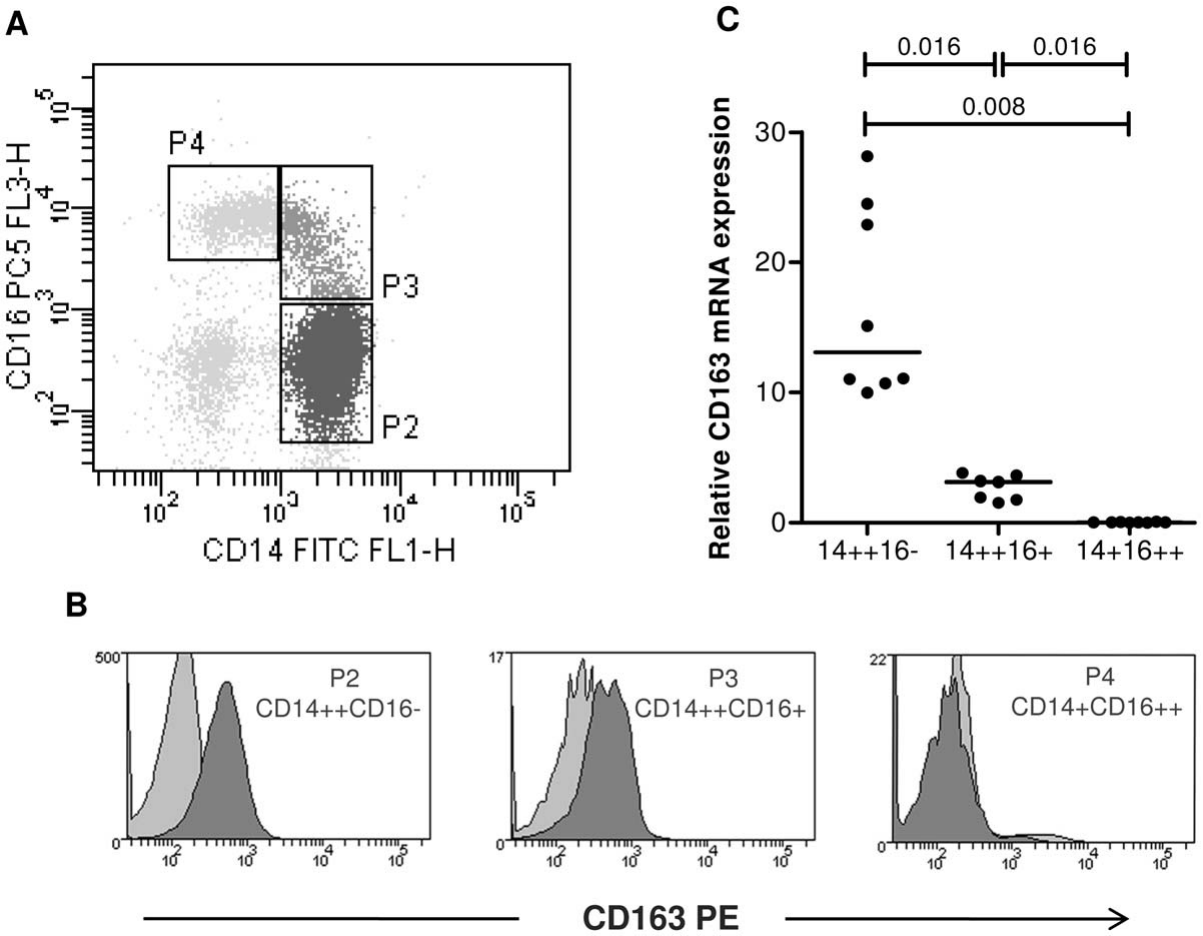
CD163 is differentially expressed on monocyte subsets at the protein and gene level. (A) Monocytes from whole blood were identified by forward and side scatter properties and expression of CD14 vs. CD16 with subsets defined as CD14++CD16− (P2, lower right), CD14++CD16+ (P3, upper right) and CD14+CD16++ (P4, upper left). (B) Expression of CD163 protein (dark grey) in each monocyte subset is shown compared to matched isotype control (light grey) in the histograms. Data illustrate results from a representative experiment. (C) CD163 mRNA was determined by Q-PCR in isolated monocyte subsets from 8 HIV-1 negative donors. Bars indicate median values. *P* values were calculated using the non-parametric, Mann-Whitney U test adjusted for multiple comparisons.

All antibodies used were purchased from BD Biosciences. An antibody cocktail composed of 8 µl anti-CD14 FITC or 4 µl anti-CD14 APC-Cy-7, 8 µl anti-CD16 PC5 and 8 µl CD163-PE (clone GHI/61; directed against domain 7 of CD163) or IgG-1 PE was placed in 5 ml polypropylene FACS tubes (BD Biosciences) and 80 µl of whole blood was added. The mixture was vortexed and incubated on ice for 30 min. Erythrocytes were lysed with 1 ml Facs Lyse (BD Biosciences) for 10 min then cells centrifuged at 500*×g* for 7 min at 4°C, with 3 ml PBS containing 1% newborn calf serum (Cosmic calf serum, HyClone) and 2 mM EDTA (wash buffer). Monocytes were initially gated using forward vs. side scatter dot plots. These events were then gated onto a CD14 vs. CD16 dot plot and monocyte subsets defined as described in figure 1A using recently defined nomenclature [47]. CD163 expression was defined as the proportion of cells within a given monocyte gate with fluorescence greater than that expressed by approximately 2% of isotype matched control cells (IgG-1 PE, BD Biosciences).

### LPS stimulation

100 µl of whole blood was washed (500*×g*, 7 min, 4°C) with 4 ml sterile wash buffer then resuspended in 1 ml supplemented Iscoves modified Dulbecco's medium (IMDM) containing 10% human serum (described below) with or without 1 µg/ml lipopolysaccharide (LPS). Cells were incubated for 2 hours at 37°C prior to washing and staining as described above.

### CD163 ELISA

Soluble CD163 from EDTA plasma samples was quantified in 2 µl of plasma using a commercial ELISA kit (BMA Biomedicals) according to manufacturer's instructions.

### Isolation of monocytes from buffy coats

Human monocytes were isolated from buffy coats of HIV-, hepatitis B virus-, hepatitis C virus-, human T cell leukemia virus-, and syphilis-seronegative donors (obtained from the Australian Red Cross Blood Services, Melbourne) by density-gradient centrifugation at 920*×g* for 20 min at room temperature, with no brake (Ficoll-Paque Plus; Amersham Biosciences), followed by counter-current elutriation (J-6M/E centrifuge equipped with a JE-5.0 rotor, Beckman Coulter). The typical purity of isolated monocytes was >90%.

### Isolation of monocyte subsets

To obtain purified CD14++CD16−, CD14++CD16+ and CD14+CD16++ monocytes for CD163 mRNA quantification, PBMC were isolated from 40 ml of freshly collected whole blood by Ficoll-Paque Plus (Amersham Biosciences) density-gradient centrifugation. Amounts not exceeding 1×10^8^ PBMC were stained with 20 µl anti-CD14 FITC, anti-HLA-DR PE and anti-CD16 PC5 for 30 min on ice before being resuspended at 1×10^7^/ml in wash buffer. HLA-DR positive cells were gated onto a CD14 vs. CD16 dot plot and cells defined as CD14++CD16−, CD14++CD16+ and CD14+CD16++ then sorted using a FACSAria (BD Biosciences) with purities of greater than 95% being achieved for all three subsets. To obtain monocyte subsets for culture *in vitro*, 5×10^7^ elutriated monocytes were stained with 20 µl CD14-FITC and 20 µl CD16-PC5 for 30 min prior to being resuspended in 5 ml sterile wash buffer then sorted as described above.

### Quantification of mRNA in monocyte subsets

To purify mRNA, purified monocyte subsets were lysed using Buffer A (100 mM Tris-HCl, pH 7.5, 500 mM LiCl, 10 mM EDTA, pH 8.0, 5 mM DTT and 1% LiDS) and mRNA captured using GenoPrep Oligo(dT) mRNA Beads as per manufacturer's instructions. Messenger RNA was eluted by resuspending washed beads in 10.4 µl DEPC-treated water and incubating at 65°C for 5 min. Complementary DNA was synthesised by incubating 11.4 µl of mRNA and Anchored-oligo(dT)18 Primer (2.5 µM, Roche) premix at 65°C for 5 min, followed by addition of 8.6 µl of master mix (1× Reaction Buffer, 20 U RNase Inhibitor, 1 mM dNTPs, 5 mM DTT and 10 U Transcriptor RT) and incubation at 55°C for 30 min then 85°C for 5 min.

For real-time PCR, 2 µl of 5-fold diluted cDNA was added to 23 µl master mix (12.5 µl SuperArray RT2 Real Time SYBR Green/Fluorescein PCR master mix (Applied Biosystems), 0.6 µM primer pair) and real time quantitative PCR (Q-PCR) analysis performed using an iCycler real-time PCR machine (BioRad). The CD163 primers were 5′-CCAGTCCCAAACACTGTCCT-3 (forward), 5′-ATGCCAGTGAGCTTCCCGTTCAGC-3′ (reverse) and amplified a 67 nucleotide sequence (GenBank accession number z22968). Relative CD163 mRNA content was determined by the comparative threshold method using GAPDH PCR as a comparator: melt curve analysis and agarose gel electrophoresis of PCR products were conducted to verify that the PCR reaction amplified a single product in each case.

### Culture of monocyte derived macrophages and monocyte subsets

Monocytes/monocyte-derived macrophages (MDM) were cultured at a concentration of 2×10^6^ cells/ml in IMDM supplemented with 2 mM L-glutamine (both Invitrogen Life Technologies) and 24 µg/ml gentamicin (supplemented IMDM) with either 10% heat inactivated pooled human serum (Australian Red Cross Blood Services, Sydney) in Teflon pots, or 10% fetal calf serum (ICP Bio) plus M-CSF (50 U/ml, R & D systems) or GM-CSF (40 ng/ml, kind gift of A. Lopez, Hanson Institute, Adelaide, Australia) in sterile 5 mm polypropylene tubes (BD Bioscience). Media was replenished with a half-media change on day 5.

### *In vitro* monocyte and macrophage phenotyping

Monocytes or macrophages (1×10^5^) were resuspended in 100 µl of either wash buffer or permeabilisation buffer (0.1% saponin freshly diluted in wash buffer) for surface or total cellular staining respectively. Cells were stained with 5 µl anti-CD163-PE or IgG-PE, or anti-CD206 (mannose receptor)–PE to confirm maturation [48], for 30 min on ice. Cells were washed once with wash buffer for surface staining or once with perm buffer and once with wash buffer for total cellular staining. Cells were then fixed with 3% formaldehyde and analysed by flow cytometry within 24 hours. Analysis was carried out using a 7 color FACSAria (BD Bioscience) with FACSDiva software, or a 3 color FacsCalibur using CellQuest software (BD Bioscience).

### Statistics

Mann-Whitney U test and Wilcoxon matched pairs test was used to test significant difference for unpaired and paired data respectively. Non-parametric Spearman correlation coefficient was used to test correlations. Statistics were generated using GraphPad Prism 5 and a *P* value lower than 0.05 was considered significant.

## Results

### CD163 is differentially expressed on monocyte subsets and can be induced by M-CSF stimulation

Expression of CD163 protein on monocyte subsets in whole blood was investigated within 4 h of venepuncture by flow cytometry. Blood was collected into K_3_EDTA-containing vacutainers since the use of both heparin and acid citrate dextran anticoagulant tubes have been reported to alter CD163 and CD16 protein expression [41], [45], [49](P. Ellery and SMC unpublished data). Monocyte subsets were defined as CD14++CD16− (P2), CD14++CD16+ (P3) and CD14+CD16++ (P4) as shown in figure 1A. CD163 expression was greatest on CD14++CD16− monocytes (median MFI [IQR] = 201.0 [176.0–374.0]), expressed at an intermediate level on CD14++CD16+ monocytes (192.0 [113.0–263.0]) and low/undetectable on CD14+CD16++ monocytes (5.0 [0–39.0]) A representative histogram of CD163 staining for each subset from a single donor is presented (Figure 1B).

To determine whether the difference in CD163 protein expression between monocyte subsets was at the level of gene expression, CD163 mRNA was quantified in the three monocyte subsets purified from eight healthy HIV-uninfected subjects (Figure 1C). CD163 mRNA expression showed a similar pattern of expression to CD163 surface expression, that is, highest in CD14++CD16− monocytes and undetectable in CD14+CD16++ monocytes. These data show that the three monocyte subsets express different amounts of CD163 on their surface and that this is consistent with the relative level of CD163 mRNA in each subset.

**Figure 2:**
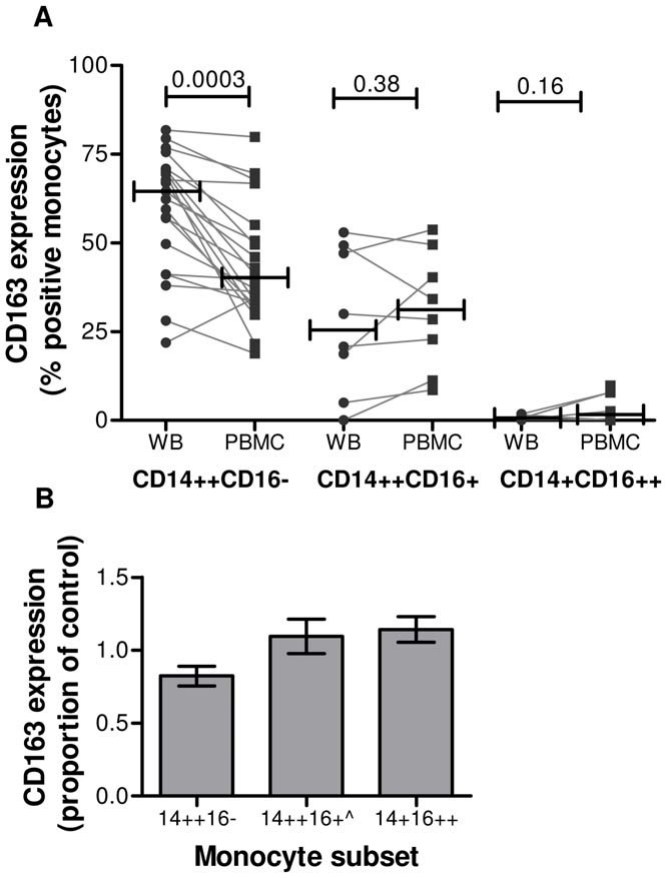
Surface CD163 expression is reduced on CD14++CD16− monocytes after density gradient separation and LPS stimulation. (A) Comparison of CD163 protein expression on the classical CD14++CD16− subset was carried out on 13 samples from HIV-1 negative donors and on all monocyte subsets from a further eight donors before and after Ficoll density gradient purification of PBMC from whole blood. Bars represent median values. Differences were analysed using the Wilcoxon matched pairs test. WB: whole blood, PBMC: peripheral blood mononuclear cells. (B) Whole blood from HIV-uninfected donors was incubated with 1 µg/ml LPS for 2 h and CD163 surface expression measured by flow cytometry. CD163 expression on each subset (mean fluorescence intensity) is plotted as the ratio of expression in treated compared to untreated monocytes. Data represent mean ± SEM for 4 independent donors. ∧ data from 1 donor was excluded from the CD14++CD16+ data set due to insufficient cell number.

To systematically investigate the effect of Ficoll density gradient isolation on monocyte subsets we performed a pair-wise comparison of the percentage of monocytes that expressed CD163 in whole blood and after PBMC preparation. CD163 expression was reduced on CD14++CD16− monocytes present in PBMC preparations compared to whole blood from the same donor by a median of 24.8% (*n* = 21, *P* = 0.0003) (Figure 2A). The proportion of CD163 positive cells in the CD14++CD16+ monocyte subset from whole blood and PBMCs were similar in eight donors tested (*P* = 0.38). These data suggest that CD163 expression on CD14++CD16− monocytes is altered by sample manipulation and that surface expression on this subset may be more labile than on other monocyte subsets.

To determine if the degree of cleavage of CD163 from the cell surface differed in different monocyte subsets, we stimulated monocytes from 4 HIV-1 negative donors with LPS to induce shedding (Figure 2B)[31], [32], [33], [34]. The mean fluorescence intensity of CD163 staining in CD14++CD16− monocytes decreased by an average of 19.7% in LPS treated monocytes compared to controls (mean MFI 503 untreated vs. 404 in LPS treated CD14++CD16− monocytes). In contrast, LPS treatment had no effect on detection of CD163 in CD14++CD16+ (mean MFI 557 untreated vs. 616 LPS treated) or CD14+CD16++ (87 untreated vs 126 LPS treated) monocyte subsets indicating that the majority of CD163 is shed from the major CD14++CD16− monocyte population after LPS stimulation.

**Figure 3:**
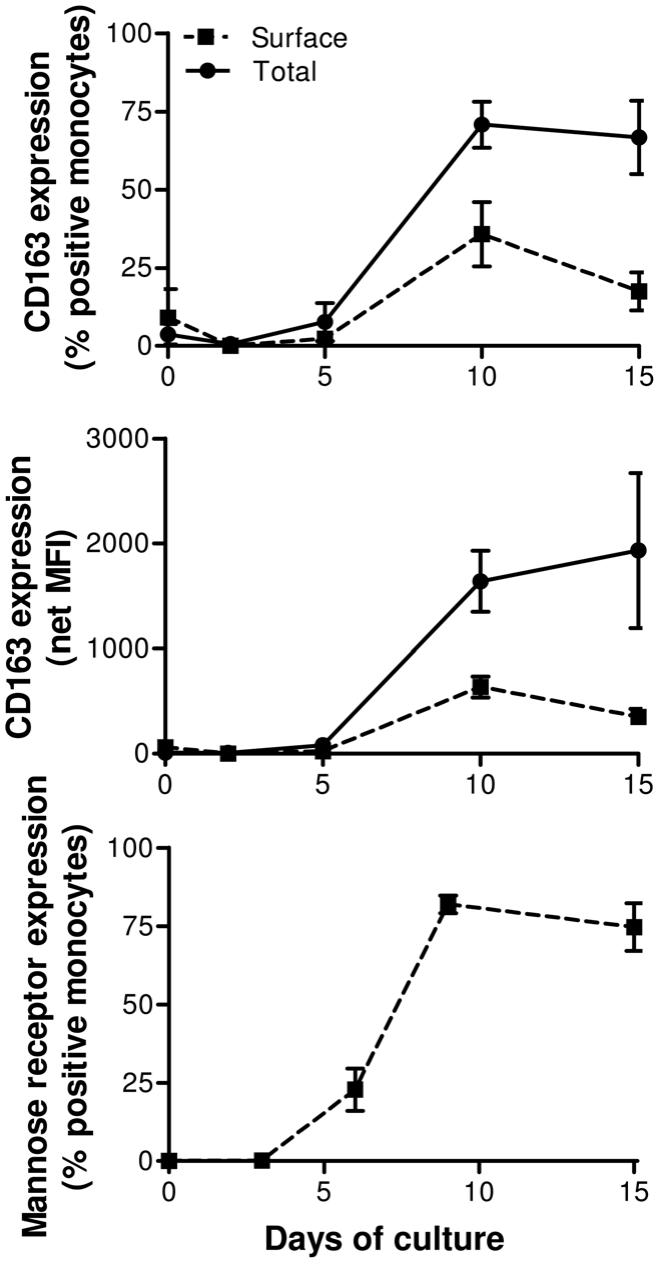
Intracellular and surface CD163 expression increases during maturation of monocytes to macrophages. Monocytes purified by countercurrent elutriation from healthy HIV-1 uninfected donors were cultured *in vitro* for the times indicated, then stained for CD163 with (solid line) and without (dashed line) permeabilisation to measure total (surface and intracellular) and surface only CD163, respectively. At the same time, cells were stained for expression of mannose receptor to confirm maturation of monocytes into macrophages. Percent of monocytes/macrophages positive for CD163 is presented in upper graph and mean fluorescence intensity of expression in middle graph. Percent of monocytes/macrophage positive for mannose receptor is presented in lower graph. Data represent mean ± SEM using 5 independent donors.

To determine how CD163 protein expression changes with differentiation of monocytes to macrophages, monocytes isolated by counter-current elutriation (*n* = 5 donors) were cultured under non-adherent conditions in IMDM supplemented with human serum but no additional growth factors. Total and surface CD163 expression and surface mannose receptor expression was measured at various times during maturation of monocytes to monocyte-derived macrophages (MDM) (Figure 3). As expected, monocytes did not express mannose receptor [48], [50], but following differentiation into macrophages its expression was detectable on the majority of cells. The frequency of CD163 expressing MDM rapidly increased after five days in culture, reaching a maximum after 10 days. A comparison of surface CD163 staining versus staining following permeabilisation showed the presence of high levels of intracellular CD163. After 10 days of culture, an average of 70.8±12.8% (mean ± standard deviation) of MDM contained intracellular/surface CD163. However, a substantial proportion (35.7±17.8%) of MDM expressing intracellular CD163 did not express detectable CD163 on the cell surface. The total MFI at the same time point was 2.5 times greater than surface alone (1639±504 vs. 635±171 fluorescence units). The fact that CD163 is predominantly expressed intracellularly in macrophages and not on the surface membrane as occurs in monocytes suggests that the function of CD163 in monocytes may differ to that of macrophages.

To address whether all three monocyte subsets are able to differentiate into CD163-expressing MDM, irrespective of initial levels of CD163 gene expression, subsets were isolated from elutriated monocytes by FACS and cultured separately in the presence of M-CSF (*n* = 4) or GM-CSF (*n* = 2) for eight days prior to determination of surface and total CD163 protein expression. Expression of CD163 protein increased in each monocyte subset to similar levels when cells were cultured with M-CSF (Table 1). Culture of monocytes from all three subsets in the presence of GM-CSF resulted in negligible expression of surface CD163. Intracellular CD163 was still detectable in an average of 32.8% of the CD14++CD16− population but only in ≤5% of CD14++CD16+ and CD14+CD16++ monocytes (Table 1). These results show that CD163 expression is highly inducible, is readily modified by macrophage growth factors and has the capacity to be expressed by all three monocyte subsets.

**Table 1:** Mean percent of cells expressing CD163 after culture of the monocytes in M-CSF or GM-CSF.

	n	CD14++CD16−	CD14++ CD16+	CD14+ CD16++
M-CSF				
Surface	4	24.7±5.5	25.2±6.2	19.0±5.7
Total	4	51.0±4.6	68.8±4.0	52.8±4.8
GM-CSF				
Surface	2	0.0±0.6	−0.3±0.4	−0.5±0.1
Total	2	32.8±14.7	5.2±6.3	1.9±1.5

Surface and total CD163 expression were measured on macrophages derived from each of the indicated monocyte subsets cultured for 8 days in the presence of M-CSF or GM-CSF.

### CD163 expression is increased on CD14++CD16+ monocytes from HIV-1 infected donors

**Figure 4:**
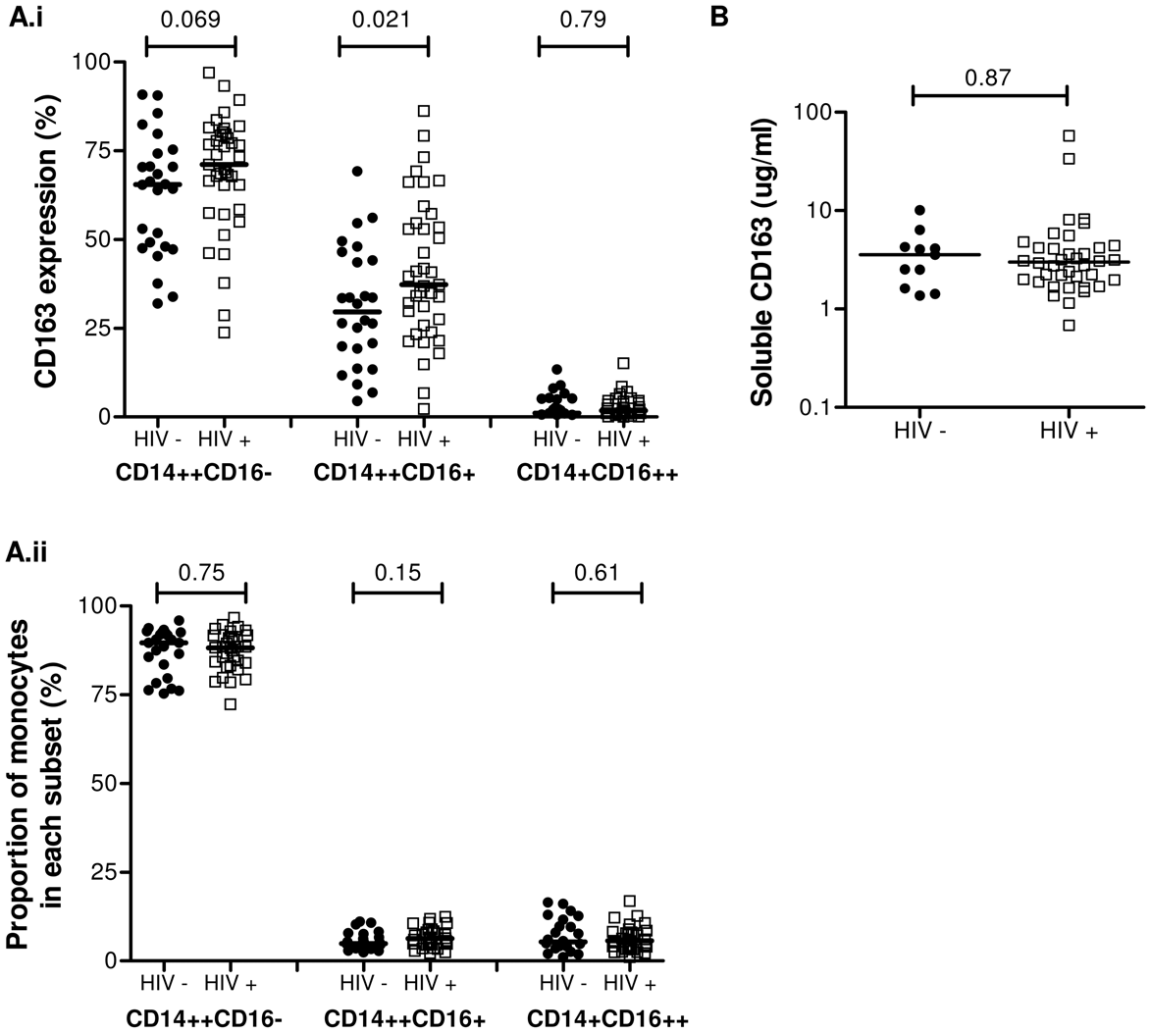
Membrane bound and soluble CD163 in monocyte subsets from HIV-1 infected donors. (A.i) CD163 expression on each monocyte subset was determined using a whole blood flow cytometric assay for HIV-1 uninfected donors (closed circles, *n* = 27) and infected donors (open squares, *n* = 38). Bars represent median values. (A.ii) The proportion of total monocytes in each of the three monocyte subsets was determined for each donor analysed in (A.i). The event count for that subset was divided by the sum of events in each subset. Bars represent median values. (B) Soluble CD163 in serum from 12 HIV uninfected donors, 38 HIV-1infected donors receiving antiretroviral therapy was determined by sandwich ELISA. Bars represent median values. *P* values were calculated using the non-parametric Mann Whitney U test.

We next compared CD163 protein expression on monocytes from HIV-infected individuals (*n* = 38), of whom 30 were currently receiving antiretroviral therapy, with 26 age-matched, healthy HIV-uninfected donors. The median CD4 T cell count and viral load for this HIV-1 cohort was 535 cells/µl and most had an undetectable viral load (<50 copies/ml) with only 9 participants having detectable viremia (Table S1). HIV-1 infected donors showed the same pattern of expression of CD163 across their monocyte subsets as uninfected donors, with the CD14++CD16− subset having the highest frequency and CD14+CD16++ monocytes the lowest frequency of CD163 expressing monocytes (Figure 4Ai). When we compared expression of CD163 on each subset in HIV-infected and uninfected donors we found significantly higher expression of CD163 on CD14++CD16+ monocytes in HIV-1 infected donors compared to HIV-1 uninfected donors (38.5% vs 29.6% respectively, *P* = 0.021). In CD14++CD16− monocytes expression of CD163 was higher in HIV-1 infected donors compared to HIV-1 uninfected donors, however, this did not reach statistical significance (72.2% vs. 65.5% respectively, *P* = 0.069).

As CD163 is shed via proteolytic cleavage [51], patients were stratified based on their receiving antiretroviral therapy regimens containing or not-containing a protease inhibitor (PI) and CD163 expression was compared on monocyte subsets. Patients treated with PIs (n = 8) expressed higher levels of CD163 on CD14++CD16− monocytes compared to those on a non-PI containing regimen, although this did not reach statistical significance (*P* = 0.056), possibly due to low sample size in the PI group. CD163 expression was not increased on CD14++CD16+ monocytes (*P* = 0.34) from patients on a PI compared to patients (n = 22) receiving non PI-containing regimens. This suggests that PIs may affect expression of CD163 on CD14++CD16− monocytes and confound measurement of CD163 on this subset in HIV patients. Exclusion of patients being treated with PIs from the analysis resulted in there being no difference in CD163 expression on CD14++CD16− monocytes of HIV+ patients compared to HIV-1 negative donors (*P* = 0.25), but a trend towards a difference in CD14++CD16+ monocytes (*P* = 0.06). There was no difference in the proportion of monocytes within each subset between HIV-1 infected and uninfected donors (Figure 4Aii) indicating that differences in CD163 expression between these two groups are not due to changes in CD16 protein expression in HIV-1 infection.

We next quantified soluble CD163 by sandwich ELISA using plasma from a randomly chosen subset of the HIV-1 uninfected donors (*n* = 11) and a cohort of HIV-1 infected donors (*n* = 38) separate from that used above (Figure 4B). In this selected group of HIV-1 infected donors median T cell count was 465 cells/µl and median viral load was 835 copies/ml with 22 out of 38 donors having a detectable viral load; 15 were not receiving antiretroviral therapy. There was no significant difference in plasma sCD163 concentrations between these two groups (median [IQR] HIV-infected = 3.0 [4.3−2.0], HIV-uninfected = 3.6 [4.3−1.6] µg/ml *P* = 0.87), nor between patients on therapy being treated with or without a protease inhibitor (*P* = 0.54).

**Figure 5:**
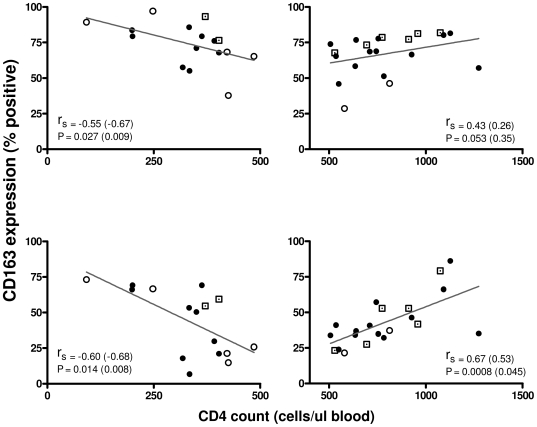
Membrane CD163 expression is related to CD4 T cell count. Frequency of CD163 positive cells in CD14++CD16− monocytes (top row) and CD14++CD16+ monocytes (bottom row) was correlated with CD4 T cell count for donors below 500 cells/µl (left column) and above 500 cells/µl (right column). Donors not currently receiving antiretroviral therapy are represented by open circles and donors receiving protease inhibitors are represented by dotted squares. r_s_ and *P* values after analysis excluding patients receiving PI are presented in parentheses after pooled data. r_s_ value was determined by Spearman correlation coefficient.

It has been reported previously in HIV-infected individuals with a CD4 count of less than 450/µl that the frequency of CD163 positive cells inversely correlated with CD4 counts [52]. We therefore examined the relationship between CD163 protein expression on both monocyte subsets and CD4 counts of the cohort, analysing expression on monocytes from patients with CD4 counts greater than or less than 500 cells/µl (Figure 5). A plot of CD163 surface expression on both the CD14++CD16− and CD14++CD16+ monocyte subsets with the patients' CD4 T cell counts at time of recruitment showed a biphasic relationship. HIV-1 infected patients with a CD4 count ≤500 cells/µl showed an inverse correlation between CD4 count and the percentage of CD14++CD16− monocytes expressing CD163 (r_s_ = −0.55, *P* = 0.027) and CD14++CD16+ monocytes expressing CD163 (r_s_ −0.60, *P* = 0.014). Conversely, HIV-1 positive patients with a CD4 T cell count >500/µl showed a positive correlation between CD163 expression and CD4 counts (r_s_ = 0.43, *P* = 0.053, for CD14++CD16− monocytes and r_s_ = 0.67, *P* = 0.0008 for CD14++CD16+ monocytes). For both CD4 T cell counts > and <500, the CD14++CD16+ subset demonstrated the strongest correlation based on both *P* and r_s_ value. Data were also examined following exclusion of results from patients being treated with PIs. For patients being treated with a non-PI containing regimen with CD4 counts less than 500/µl the correlation between CD4 count and surface CD163 expression was strengthened for both CD14++CD16− and CD14++CD16− monocytes (r_s_ = −0.67, *P* = 0.009 and r_s_ = −0.68, *P* = 0.008 respectively). There was no correlation between patient viral load and surface CD163 on either monocyte subset (data not shown).

Examination of sCD163 levels and CD4 T cell count showed a weak correlation (r_s_ = 0.34), such that patients with low CD4 T cell counts had low sCD163 levels (Table S2). Further analysis of both sCD163 and surface CD163 expression compared to CD4 cell count in various clinical groups is presented in supplementary data table S2.

## Discussion

In this study we have used whole blood flow cytometric phenotyping assays and quantitative real time PCR to show that CD163 is expressed in high levels on the classical CD14++CD16− monocytes, intermediate levels on CD14++CD16+ monocytes and not at all on non-classical CD14+CD16++ monocytes. We show that CD163 surface expression is decreased selectively on CD14++CD16− monocytes during monocyte purification which, together with variable antibody affinities [46], explanat the discrepancies in the literature regarding monocyte expression of CD163 [25], [52]. We further show that all monocyte subsets induce comparable CD163 expression following differentiation to type 2 (anti-inflammatory) macrophages in the presence of M-CSF which suggests that CD163 positive macrophages are not derived exclusively from CD163 expressing monocytes. Finally, we show that CD163 is elevated on monocytes from HIV-1 infected individuals. These data support the hypothesis that CD163 could serve as a biomarker for immune activation and/or resolution of inflammation in HIV-infected individuals in the HAART era [5], [6], [7], [13], [32], [39], [53].

There are several conflicting reports regarding CD163 protein expression on monocyte subsets. The reported frequency of CD163 positive monocytes from healthy individuals has ranged from undetectable to as high as 100% [54], [55]. CD163 protein expression on monocyte subsets has also previously been reported to be highest on CD16+ monocytes [25]. We demonstrate that CD163 is detectable by flow cytometry on the majority of CD14++CD16− monocytes and in approximately half of CD14++CD16+ monocytes. These findings are similar, albeit at a lower level, to those reported by Monisuszko et al. [20]. In contrast to several reports [20], [25], however, we found surface CD163 is undetectable in CD14+CD16++ monocytes *ex vivo*. These findings are reflected by CD163 gene expression measured by real time RT-PCR. The differential expression of CD163 between subsets may reflect distinct monocyte subset functions, with the absence of CD163 on CD14+CD16++ monocytes consistent with their pro-inflammatory role [56], [57].

The conflicting results reported in the literature regarding CD163 expression on monocyte subsets are likely to be due to a range of technical issues. These include anticoagulant type [52], [58], [59], antibody used (relating to access to epitope as well as extracellular calcium dependence [46]), analysis of PBMC prepared using Ficoll density gradient isolation [52], [58], [59] and monocyte culture prior to analysis [25], [60]. These incongruities highlight the need for techniques of measuring CD163 expression that involve the least amount of sample manipulation, such as using whole blood techniques.

Examination of the effect of sample manipulation on expression of CD163 on the surface of monocytes confirmed observations by others [45]. However, we further show that whilst CD163 expression was significantly decreased on CD14++CD16− monocytes following sample manipulation, expression on CD14++CD16+ monocytes was not changed following Ficoll density gradient centrifugation. This suggests that CD163 is more labile on the former subset. To investigate this observation we induced CD163 shedding by LPS and found similarly reduced CD163 surface expression on CD14++CD16− monocytes but comparable levels on CD14++CD16+ monocytes compared to controls.

CD163 surface expression has been shown to be altered by cleavage of CD163 mediated by a metalloproteinase, specifically ADAM17 (a disintegrin and metalloproteinase domain 17) [51], rather than loss of epitopes due to conformational changes or protein internalisation [31], [32], [33], [34]. Monocyte subsets differentially express metalloproteinases [61], [62]. This most likely accounts for the different degree of CD163 shedding between the subsets demonstrated here, however, ADAM17 expression has not yet been characterised on monocyte subsets. Our observations showing an LPS-induced decrease in CD163 expression on the classic CD14++CD16− monocytes but not CD14++CD16+ monocytes suggest that the majority of CD163 found as sCD163 in plasma originates from CD14++CD16− monocytes.

CD163 expression on monocytes and macrophages is readily modified by immune factors such as cytokines, inflammatory mediators and bacterial components. We showed that when monocytes are matured into macrophages CD163 expression is upregulated and that a significant proportion of CD163 is found intracellularly. This intracellular pool of CD163 may be maintained to enable rapid secretion upon stimulation, or may indicate constitutive endocytosis [63]. To investigate the potential for each subset to express CD163, isolated monocyte subsets were differentiated [64] in the presence of GM-CSF and M-CSF. Using this experimental protocol circumvents the problems resulting from the low viability of CD16+ monocytes cultured in the absence of added cytokines, and provides additional information regarding pro-inflammatory type 1 and anti-inflammatory type 2 macrophages [64]. Expression of CD163 on monocyte-derived macrophages from each subset was low after culturing in the presence of GM-CSF, supporting previous observations [64], [65]. In contrast, MDM derived from monocyte subsets cultured in the presence of M-CSF upregulated CD163 in each subset to comparable levels. These data indicate that despite undetectable mRNA levels in circulating CD14+CD16++ monocytes CD163 expression can be induced by M-CSF to levels comparable to the CD14++CD16− population.

It is uncertain whether different monocyte subsets have different differentiation fates in vivo. Our findings have implications regarding the origin of perivascular macrophages in the brain which express both CD16 and CD163. Several reports have suggested that CD14+CD16+CD163+ monocytes circulating in the peripheral blood are potentially the precursors of perivascular macrophages due to the similarity in phenotype [41], [52]. Given that CD16 is induced during monocyte maturation [66] and that we have shown that CD163 can be expressed in any monocyte subset upon stimulation, these markers are not reliable in predicting the origin of perivascular macrophages. As monocyte subsets can be matured into cells with distinct functional characteristics in vitro [67], further work is required to determine if, despite similar CD163 expression, type II macrophages derived from different subsets have differences in immune function such as bacterial phagocytic or T cell stimulating ability.

Our data show that the frequency of CD14++CD16−CD163+ and CD14++CD16+CD163+ monocytes from HIV-infected individuals was increased relative to age-matched, HIV-uninfected individuals, with differences in the latter subset reaching statistical significance. There was no significant change in the proportion of each monocyte subset to total monocytes from HIV-1 infected donors, the majority of whom were on HAART with an undetectable viral load, compared to HIV-uninfected controls (similar to our earlier observations [68]) and so this finding was not due to upregulation of CD16 during HIV-1 infection. Comparison of CD163 expression on monocytes from patients receiving or not receiving a PI-containing regimen suggests that protease inhibitors may selectively decrease cleavage of CD163 from CD14++CD16− monocytes, accounting for the trend to increased CD163 expression seen in HIV-infected versus HIV-1 uninfected donors. In their study, Fischer-Smith et al. did not demonstrate increased CD163 expression on CD14++CD16− monocytes from HIV-1 infected donors [52]. This may be due to a range of factors, including no patients receiving PI therapy in their cohort, as well as examination of phenotype on PBMC post Ficoll isolation which we have shown to selectively result in loss of CD163 from CD14++CD16− monocytes. We did not find any association between receiving a PI-containing regimen and levels of sCD163 in plasma, but this is presumably due to the low numbers of our patients receiving PIs. Direct comparison of CD163 expression on CD14++CD16− monocytes and soluble CD163 levels in single donors from a larger cohort may uncover a connection between these parameters.

We observed a weak overall correlation between sCD163 levels and CD4 T cell counts with no correlation between sCD163 and HIV RNA. Previously, Fischer-Smith et al. reported an inverse correlation between frequency of CD163+CD14++CD16+ monocytes and CD4 T cell count in HIV-1 positive donors with counts below 450 cells/µl [52]. We stratified our surface expression data based on the commonly used and thus more clinically relevant CD4 cutoff of 500 cells/µl and correlated CD163 expression with CD4 counts. Whilst we found a similar negative correlation between CD163 positive monocyte frequency and CD4 counts in patients with low CD4 counts (for both CD14++CD16+ monocytes as well as CD14++CD16− monocytes), our data also show a positive correlation in both monocyte subsets in donors with T cell counts above 500 cells/µl. These observations were similar when we stratified our patients using the same CD4 cutoff as Fischer–Smith and colleagues. These findings are intriguing and suggest alteration in CD163 expression on CD14++CD16+ monocytes in HIV-infected individuals may reflect either HIV- or immune–related influences on CD163 gene and protein expression and shedding. This might include an altered cytokine milieu in individuals at different stages of disease e.g. raised plasma IL-10 and cortisol [23], [25], [54], [69], [70] (reviewed [71]), defects in cleavage of CD163 as a consequence of HIV-1 therapy, contribution from comorbid illness such as cardiovascular disease, or possibly alterations in gut lymphoid mass with resulting bacterial translocation [72], [73]. To fully elucidate the factors that modulate CD163 expression in HIV-1 patients, larger cohorts stratified on therapy type and comorbid disease are required.

Several studies have investigated changes in both membrane bound and soluble CD163 in response to acute illness. Examination of sCD163 levels in hospitalised patients demonstrated that sCD163 is an acute phase marker which increases in response to bacterial infection [5], [8], [32], [35], [36], [37], [74]. We did not, however, find such a correlation in our cohort of HIV-1 infected donors. Administration of endotoxin to healthy human subjects results in a rapid increase in sCD163 levels with concomitant loss of membrane expression on monocytes [33]. Given that LPS levels are elevated in plasma of HIV-infected patients, even in those whose viremia is suppressed by ART [72], [73], we predicted that CD163 expression on CD14++CD16− monocytes would be decreased and sCD163 levels increased. However, we show that membrane bound CD163 in fact increases and there is no detectable change in sCD163 levels in HIV-1 infection. Recent investigation into the effect of chronically raised LPS serum levels on monocyte activation in HIV-1 infected people demonstrated that monocytes become resistant to the stimulating effect of LPS [75]. Monocyte tolerance to endotoxin may explain why we did not find increased sCD163 plasma levels and the typical changes in CD163 expression seen after acute exposure to endotoxin *in vivo* [33]. As CD163 is involved in immune resolution, abrogated CD163 responses to inflammatory stimuli may contribute to the observed chronic immune activation/inflammation that is characteristic of HIV infection. Further investigation into the effect of chronic bacterial translocation on CD163 expression and shedding in HIV-1 infection, and correlation with other markers of inflammation, may uncover a better understanding of chronic immune activation in HIV-1 infection.

The development of biomarkers that reflect chronic inflammation and/or immune activation is critical to ensure better long term outcomes for people with HIV-1. CD163 shows potential promise as a biomarker of monocyte/macrophage activation, but our data suggest that a complex relationship exists between CD163 monocyte expression and shedding on the one hand and HIV disease progression on the other. Further studies are warranted to define these relationships before CD163 could be used as a prognostic indicator of HIV-1 related co-morbidities.

In the early days of antiretroviral therapy, goals for treatment focused on maintaining life. Due to the success of combination antiretroviral therapy focus has shifted to maintaining the health of HIV-infected individuals. Current antiretroviral therapy, despite effective viral suppression, does not eliminate immune activation and chronic inflammation persists, leading to an increased incidence of age-associated inflammatory diseases and a shortened lifespan. As CD163 is involved in immune resolution and protection against the development of atherosclerotic plaques, understanding the changes in regulation of CD163 in HIV-1 is important in addressing comorbid diseases caused by chronic immune activation.

## Supporting Information

Table S1 Virological and immunological characteristics and antiretroviral therapy of HIV-1 infected donors at time of participation.
(DOC)

Table S2 Correlation of membrane bound CD163 on monocyte subsets and soluble CD163 in plasma stratified by clinical parameter.
(DOC)
