# Supplementary material for: Differential Expression of CD163 on Monocyte Subsets in Healthy and HIV-1 Infected Individuals
Source: PLoS One. 2011 May 20;6(5):e19968. doi: 10.1371/journal.pone.0019968 (PMC3098854; doi:10.1371/journal.pone.0019968)
Supplement: Table S1 — Virological and immunological characteristics and antiretroviral therapy of HIV-1 infected donors at time of participation. (DOC) [file pone.0019968.s001.doc]

Supplementary Table 1: Virological and immunological characteristics and antiretroviral therapy of HIV-1 infected donors at time of participation

| **Pt No.** | **Viral Load** | **CD4 T cell Count** | **Therapy** |
| --- | --- | --- | --- |
| **Surface CD163** | |  |  |
| 1 | <50 | 550 | NRTI/NtRTI^1^ x 2, NNRTI^2^ |
| 2 | <50 | 403 | NRTI/NtRTI x 2, FI^3^ PI^4^ |
| 3 | <50 | 710 | NRTI/NtRTI x 2, NNRTI |
| 4 | 1700 | 200 | NRTI/NtRTI x 2, NNRTI |
| 5 | 44900 | 92 | None |
| 6 | <50 | 333 | NRTI/NtRTI x 3 |
| 7 | <50 | 911 | NRTI/NtRTI x 2, PI |
| 8 | <50 | 1075 | NRTI/NtRTI x 2, PI |
| 9 |  |  | No Record |
| 10 | <50 | 350 | NRTI/NtRTI x 2, NNRTI |
| 11 | <50 | 507 | NRTI/NtRTI x 2, NNRTI |
| 12 | <50 | 1092 | NRTI/NtRTI x 3 |
| 13 | 12800 | 813 | None |
| 14 | <50 | 774 | NRTI/NtRTI x 2, PI |
| 15 | 45200 | 425 | None |
| 16 | 6700 | 485 | None |
| 17 | <50 | 392 | NRTI/NtRTI x 2, NNRTI |
| 18 | 24600 | 422 | None |
| 19 | <50 | 530 | NRTI/NtRTI x 2, PI |
| 20 | <50 | 927 | NRTI/NtRTI x 2, NNRTI |
| 21 | <50 | 640 | NRTI/NtRTI x 2, PI |
| 22 | <50 | 199 | NRTI/NtRTI x 2, NNRTI |
| 23 | <50 | 1273 | NRTI/NtRTI x 2, NNRTI |
| 24 | <50 | 535 | NRTI/NtRTI x 2, NNRTI |
| 25 | <50 | 635 | NRTI/NtRTI x 2, NNRTI, PI, INTI^5^ |
| 26 | <50 | 958 | NNRTI, PI |
| 27 | <50 | 744 | NRTI/NtRTI x 2, NNRTI |
| 28 | <50 | 695 | NRTI/NtRTI x 2, PI |
| 29 | <50 | 334 | NRTI/NtRTI x 2, NNRTI |
| 30 | <50 | 754 | NRTI/NtRTI x 2, NNRTI |
| 31 | <50 | 318 | NRTI/NtRTI x 2, NNRTI x 2 |
| 32 | <50 | 403 | NRTI/NtRTI x 2, CRA^6^ |
| 33 | 71100 | 580 | None |
| 34a | 3880 | 1126 | NRTI/NtRTI x 2, NNRTI, INTI |
| 35a | <50 | 783 | None |
| 36a | 16200 | 248 | None |
| 37 | <50 | 371 | NRTI/NtRTI x 2, PI |
| 38a | <50 | 363 | NRTI/NtRTI x 2, NNRTI, INTI |
| **Soluble CD163** | |  |  |
| 34b | 16200 | 248 | None |
| 35b | 12400 | 63 | None |
| 36b | <50 | 1426 | NRTI/NtRTI x 2, NNRTI, INTI |
| 37 | <50 | 371 | NRTI/NtRTI x 2, PI |
| 38b | >100000 | 48 | NRTI/NtRTI x 3, PI |
| 39 | <50 | 375 | NRTI/NtRTI x 3, PI x 2, INTI |
| 40 | <50 | 372 | NRTI/NtRTI x 2, NNRTI |
| 41 | <50 | 433 | NRTI/NtRTI x 2, NNRTI |
| 42 | 4500 | 35 | NRTI/NtRTI x 3, FI, PI |
| 43 | <50 | 616 | NRTI/NtRTI x 3, NNRTI |
| 44 | 21600 | 907 | None |
| 45 | 32900 | 530 | None |
| 46 | <50 | 259 | NRTI/NtRTI, NNRTI, PI |
| 47 | 8400 | 1043 | NRTI/NtRTI, PI, FI |
| 48 | 42200 | 598 | None |
| 49 | >100000 | 580 | None |
| 50 | 200 | 494 | NRTI/NtRTI x 2, NNRTI |
| 51 | <50 | 180 | NRTI/NtRTI x 2, PI |
| 52 | >100000 | 491 | None |
| 53 | 42200 | 468 | None |
| 54 | <50 | 367 | NRTI/NtRTI x 2, NNRTI |
| 55 | <50 | 467 | NRTI/NtRTI x 2, PI |
| 56 | 31600 | 699 | None |
| 57 | <50 | 1254 | NRTI/NtRTI x 2, PI |
| 58 | 37300 | 697 | None |
| 59 | 600 | 218 | NRTI/NtRTI x 3, FI, INTI, PI |
| 60 | <50 | 957 | NRTI/NtRTI x 2, NNRTI |
| 61 | <50 | 596 | NRTI/NtRTI x 2, NNRTI |
| 62 | 600 | 356 | NRTI/NtRTI x 4 |
| 63 | <50 | 464 | NRTI/NtRTI x 2, NNRTI |
| 64 | 47600 | 354 | None |
| 65 | 6600 | 472 | None |
| 66 | >100000 | 383 | None |
| 67 | <50 | 317 | NRTI/NtRTI x 2, NNRTI, PI |
| 68 | 10500 | 75 | None |
| 69 | 1070 | 636 | NRTI/NtRTI x 2, PI |
| 70 | <50 | 837 | NRTI/NtRTI x 2, NNRTI |
| 71 | >100000 | 57 | None |
|  |  |  |  |

^1^ Nucleoside/nucleotide reverse transcriptase inhibitor

^2^ Non-nucleoside reverse transcriptase inhibitor

^3^ Fusion inhibitor

^4^ Protease inhibitor

^5^ Integrase inhibitor

^6^ CCR5 receptor antagonist

a, b Several donors had blood collected at different time points; a is first collection and b is second collection for the same donor. Clinical data is specific to the time of collection.
