# Supplementary material for: Differential Expression of CD163 on Monocyte Subsets in Healthy and HIV-1 Infected Individuals
Source: PLoS One. 2011 May 20;6(5):e19968. doi: 10.1371/journal.pone.0019968 (PMC3098854; doi:10.1371/journal.pone.0019968)
Supplement: Table S2 — Correlation of membrane bound CD163 on monocyte subsets and soluble CD163 in plasma stratified by clinical parameter. (DOC) [file pone.0019968.s002.doc]

**Supplementary Table 2: Correlation of membrane bound CD163 on monocyte subsets and soluble CD163 in plasma stratified by clinical parameter.**

|  | **Monocyte surface expression** | | | | | |  | **Soluble CD163** | | | |
| --- | --- | --- | --- | --- | --- | --- | --- | --- | --- | --- | --- |
| **Sample** |  | **14++16-** | | **14++16+** | |  | | |  |  |  |
| **group** | ***n*** | **P** | **r_s_** | **P** | **r_s_** |  | | | ***n*** | **P** | **r_s_** |
| **All donors** | 37 | 0.36 | -0.16 | 0.74 | 0.11 |  | | | 35 | 0.045 | 0.34 |
| **CD4 <500** | 16 | 0.027 | -0.55 | 0.014 | -0.60 |  | | | 23 | 0.08 | 0.37 |
| **CD4 <600** | 21 | 0.001 | -0.65 | 0.021 | -0.50 |  | | | 26 | 0.019 | 0.46 |
| **No ART** | 7 | 0.036 | -0.78 | 0.40 | -0.36 |  | | | 11 | 0.022 | 0.68 |

Correlation of CD163 surface expression on CD14++CD16- and CD14++CD16+ monocytes (left panel) or soluble CD163 (right panel) to donor CD4 count stratified by clinical parameters; all donors tested, donors with CD4 count less than 500 and 600 cells/µl and donors not receiving antiretroviral therapy. Correlation was determined using Spearman correlation coefficient. Columns are sample size (*n*), P value of correlation (P) and correlation coefficient (r_s_). For both cell associated and soluble CD163 the strongest correlation with CD4 T cell count was found for donors with T cell counts below 600 cells/µl.
